# Supplementary figures and images for: Low oxygen alters mitochondrial function and response to oxidative stress in human neural progenitor cells
Source: PeerJ. 2015 Dec 10;3:e1486. doi: 10.7717/peerj.1486 (PMC4690376; doi:10.7717/peerj.1486)

act.Casp3/7<sup>+</sup>

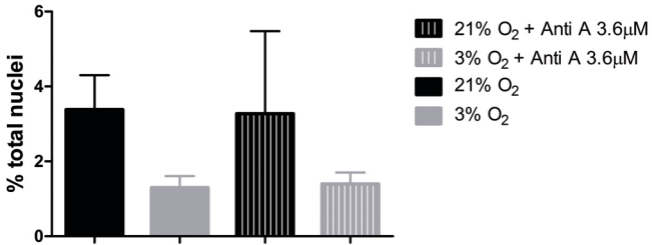

Supplement: Figure S1 — NPCs grown in either physiological oxygen concentrations (gray bars), or in normoxia (black bars), do not show differences in apoptosis after treated with 3.6 µM antimycin A for 40 min. [file peerj-03-1486-s001.pdf]

A

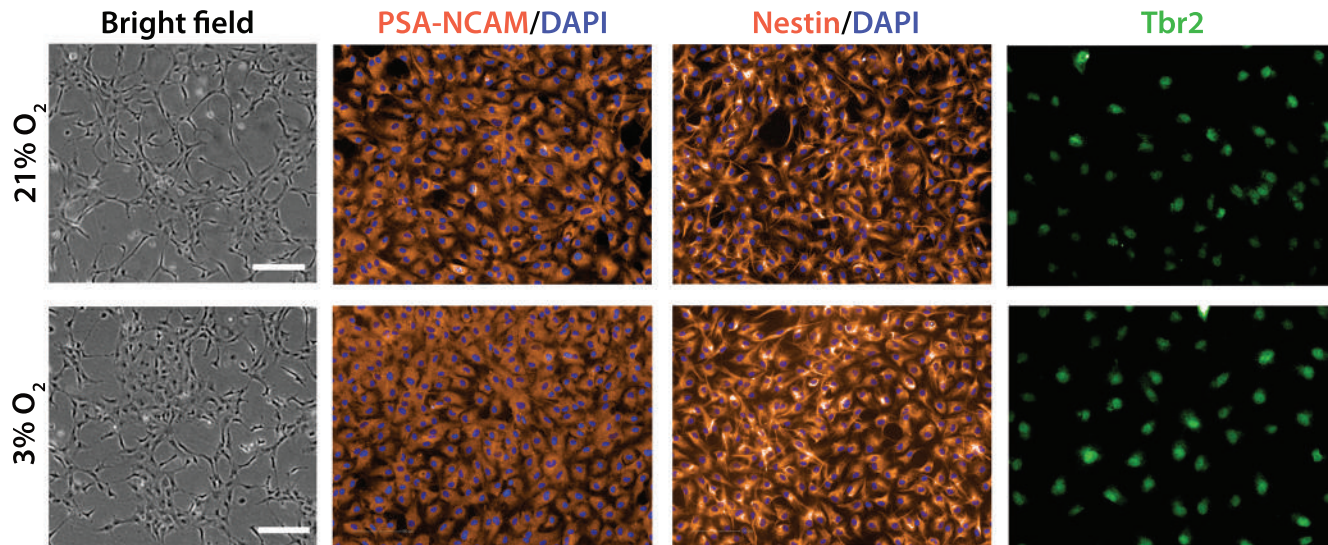

B

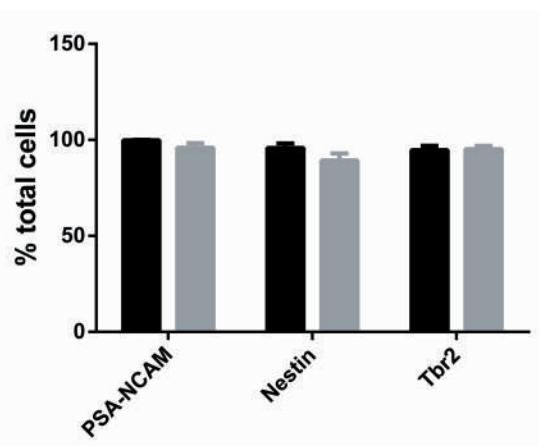

C

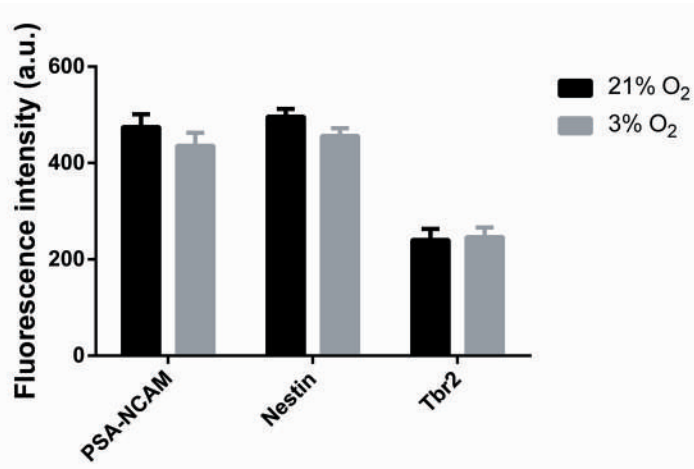

Supplement: Figure S2 — NPCs grown in physiological oxygen concentrations show the same morphology, and expression of characteristic markers, as those propagated in normoxia. Bright field and immunofluorescence images are displayed in (A), and quantification of classic NPC markers, such as PSA-NCAM, Nestin and Tbr2 are shown as for total amount of stained cells in (B), and as their fluorescence intensity in (C). Scale bars: 200 µm for bright field images, 100 µm for PSA-NCAM and Nestin, and 50 µm for Tbr2. [file peerj-03-1486-s002.pdf]
